# Health education programs for improving men’s engagement with health services in low- to middle-income countries: a scoping review protocol

**DOI:** 10.1186/s13643-019-1266-3

**Published:** 2020-01-07

**Authors:** Tafadzwa Dzinamarira, Desmond Kuupiel, Tivani Phosa Mashamba-Thompson

**Affiliations:** Department of Public Health Medicine, School of Nursing and Public Health, University of KwaZulu-Natal, Durban, 4001 South Africa

**Keywords:** Health education programs, Men, Low- to middle-income countries

## Abstract

**Background:**

Health education programs (HEPs) have been documented to increase individuals’ awareness toward their health and improve health outcomes. Given the reported poor health seeking behavior among men in low- to middle-income countries (LMICs), it is crucial for HEPs to be targeted toward men in order to improve health outcomes. Here, we outline a protocol for a scoping review aimed at mapping literature on HEPs for men in LMICs in order to reveal gaps to guide future research and practice.

**Methods:**

We will conduct a scoping review with guidance from the Arksey and O’Malley framework (Journal of Social Research Methodology 8(1):19–32, 2005), further enhanced by Levac et al. (Implementation Science 5(1):69, 2010). We will conduct a comprehensive keyword search for relevant studies presenting evidence on HEP for men in LMICs from PubMed, Google Scholar, EBSCOhost, and WEB of Science databases. In addition, we will search for relevant gray literature, dissertations, and theses from university repositories as well as international organizations such as the World Health Organization (WHO). We will include articles reporting evidence on health education programs for men in LMICs and published between January 2000 and March 2019. We will employ NVIVO version 12 software package to extract the relevant outcomes from the included articles using content thematic analysis. We will conduct quality appraisal of the included articles using the mixed methods appraisal tool (MMAT) 2018 version.

**Discussion:**

We anticipate to find relevant studies reporting on health education programs for men in LMICs. The findings of this review will guide further implementation research on health education programs for men in LMICs. The results of the proposed scoping review will be disseminated electronically, in print, and through conference presentation as well as key stakeholder meetings.

## Background

In most parts of the world, health outcomes among boys and men continue to be substantially worse than among girls and women [1, 2]. Available evidence shows men to have an average of 5–7 year lower life expectancy than women [3]. Literature on the social determinants of health and men’s poorer survival rates reflect several factors contributing toward low life expectancy, which include behavior associated with male norms of risk-taking and paradigms related to masculinity [1, 4, 5]. Men’s health has become of major concern to health policymakers and healthcare providers especially in LMICs, where the resources are limited and burden to healthcare systems are a reality [6, 7]. Evidence shows that males in low- to middle-income countries (LMICs) are not accessing health services as much as their female counterparts resulting in worse outcomes which include mortality [1, 2, 8]. Health education programs (HEPs) have been recommended to address poor health seeking behavior among key populations including men [9, 10]. HEPs are a form of health promotion through health literacy and are immensely beneficial in keeping people alive by providing valued services to populations, information on combating various diseases and promoting health awareness [11].

Ensuring healthy lives and promoting well-being for all at all ages is one of the key agendas adopted by the UN General Assembly for 2030 [12]. Gender-specific strategies for care delivery, health education, and research are key to achieving these goals, particularly in disease-burdened LMICs [9, 13–15]. A 2007 World Health Organization (WHO) review of interventions designed to involve men and boys in achieving gender equality and better health concluded that well-designed male involvement interventions would be more effective at improving men’s attitudes and behaviors toward sexual and reproductive health and other health conditions [16]. However, progress toward engaging men in participation in health programs has been slow [17].

HEPs have been documented to enhance health of various population groups and to be effective in improving health outcomes in sexually transmitted diseases and chronic conditions such as Parkinson’s disease [18–21]. However, the level of evidence on men’s engagement with HEPs in LMICs is not clear. The main objective of the scoping review is to map literature on HEPs for improving men’s engagement with health services in LMICs. It is anticipated that findings from this study enable the researchers to identify gaps in the subject matter and guide future research to bridge the gaps toward improved health outcomes for men in LMICs. The results of this study will also guide HEP implementers on designing gender-specific programs to help improve men’s engagement with health services in LMICs.

## Methodology

By systematically searching, selecting, and synthesizing existing knowledge, we will use a scoping review to synthesize knowledge in order to answer our research question [22]. The review will be guided by the Arksey and O’Malley framework [23], further enhanced by Levac et al. [24], which entails identification of the research question, identification of relevant studies, study selection, charting the data, and collating, summarizing, and reporting of the results. We will also appraise the quality of the included studies as recommended by Levac et al. [24].

### Eligibility of the research question for a scoping review

Our research question is: What is the evidence on HEPs for enabling men’s engagement with health services in LMICs?

We have used the Population, Concept, and Context (PCC) framework to determine the eligibility of our research question for a scoping review study (Table 1).

**Table 1:** Framework for determining the eligibility of the research question

Population	Human participants; male sex; 15 years and older in low- to middle-income countries as classified by World Bank [25]
Concept	Any health education program for men implemented between January 2000 and March 2019
Context	Health service engagement

### Identifying relevant studies

We will conduct a comprehensive search of relevant articles from the following electronic databases for articles published between January 2000 and March 2019: Google Scholar, PubMed, EBSCOhos, and WEB of Science. In addition, we will search on ResearchGate as well as gray literature from university dissertations and theses from institutional repositories, government, and international organizations’ reports such as the WHO. We will search for randomized controlled trials, non-randomized controlled trials, and observational studies that report evidence on HEPs for men engagement in health services. Review articles will be excluded. Medical Subject Headings (MeSH) terms will be included during the search for relevant articles. After searching, the studies will be screened against the inclusion and exclusion criteria. The search terms will include “health,” “education,” “program,” and “men”. Boolean terms, AND and OR, will be used to separate the keywords. The reference lists of included articles will also be searched for relevant studies. We will adapt search strategy to suit each database. Each search will be documented in detail showing the keywords, date of search, search engine, and number of publications retrieved.

We have conducted a pilot search in Google Scholar to demonstrate feasibility of answering our research question using a scoping review method. The results of our pilot search are presented in Table 2.

**Table 2:** Results of pilot search in Google Scholar

Keywords search	Date of search	Search engine used	Number of publications retrieved
((low[All Fields] AND middle[All Fields] AND (“income”[MeSH Terms] OR “income”[All Fields]) AND countries[All Fields]) AND (“men”[MeSH Terms] OR “men”[All Fields])) AND ((“health education”[MeSH Terms] OR (“health”[All Fields] AND “education”[All Fields]) OR “health education”[All Fields]) AND programs[All Fields])	20 March 2019	Google Scholar	1790

### Study selection

Selection criteria were developed in order to ensure that relevant studies on health programs for men in LMICs will be included.

#### Inclusion criteria

To be included, articles must meet the following criteria:

- Reporting evidence on health education programs for men aged 15 and older in LMICs
- Reporting evidence on health education programs improving men’s engagement in health services in LMICs
- Published between January 2000 and March 2019

#### Exclusion criteria

We will exclude articles guided by the following exclusion criteria:

- Articles reporting evidence on health education programs for women and children
- Articles reporting evidence of health education programs for men in high-income countries and upper LMICs
- Articles published before January 2000 and after March 2019

**Fig 1.**
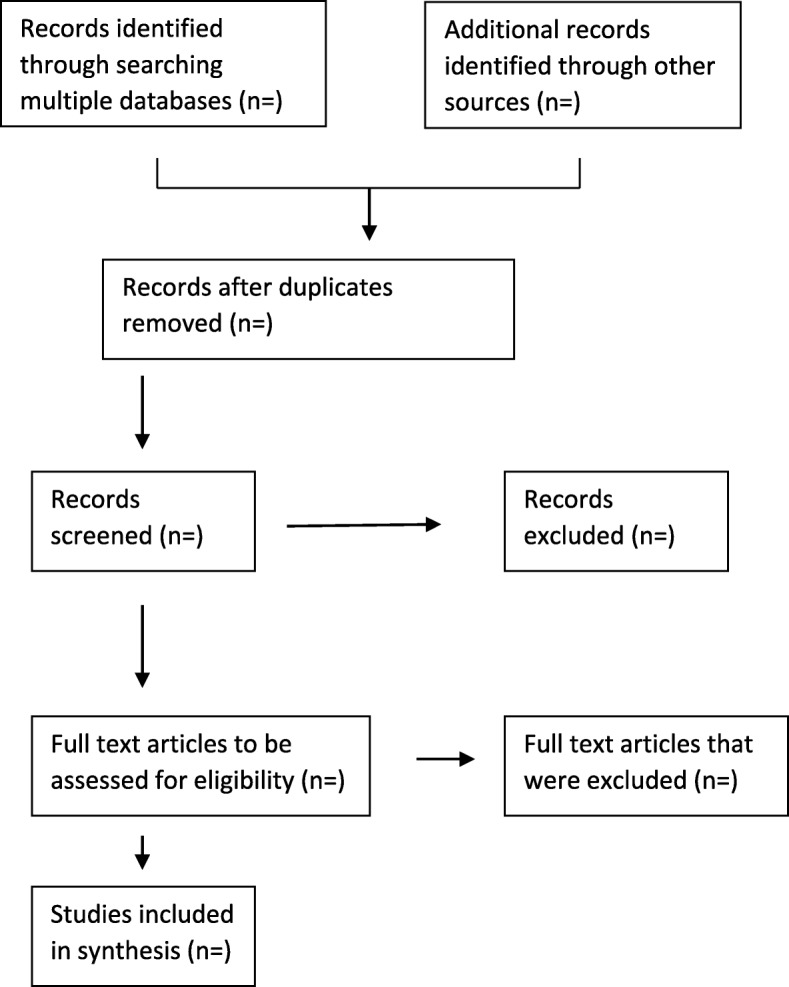
PRISMA extension for scoping reviews, 2018 flow diagram [26]

Following database search, retrieved articles will be screened in three stages. First, the principal investigator (PI) will screen titles of the articles retrieved from database search. An Endnote library using the Endnote X8 will be created, and all eligible articles following title screening will be exported into the library. Second, two independent trained reviewers will screen abstracts in parallel. Discrepancies between reviewers’ response will be resolved through a discussion by the review team. Third, all eligible full articles following abstract screening will also be screened by two independent trained reviewers. We will calculate the kappa statistic to determine the level of agreement between the two reviewers. A kappa statistic of > 0.61 will be considered acceptable agreement. Discrepancies in reviewers’ responses following full article screening will be resolved by inviting a third screener. We will report the screening results following the Preferred Reporting Items for Systematic Reviews and Meta-Analyses (PRISMA) guidelines [26] (Fig. 1).

### Charting the data

We developed a data charting form for extracting relevant data to characterize included studies (Table 3). Two independent reviewers will pilot the data charting form using a random sample of 10 included studies for consistency. We will modify the data extraction form as required based on feedback from the two reviewers. We will also constantly update the data charting form throughout the duration of the study.

**Table 3:** Data charting form

Author and publication year	
Study title	
Total participants	
Percentage of men	
Age of participants	
Study settings	
Geography (where was the study area)	
Type of health education program carried out	
Study design	
Study findings	
Conclusions	

### Collating, summarizing, and reporting results

We will employ NVivo version 12 to conduct content thematic analysis of the included studies. We will present a narrative account of the findings presenting the main concepts from the included articles in line with our research question.

### Quality appraisal

The mixed method appraisal tool (MMAT) version 2018 will be used to assess the quality of the included articles [27]. The tool will be used to examine the aim of the study, adequacy and methodology, study design, participant recruitment, data collection, data analysis, presentation of findings, and authors’ discussions and conclusions. The MMAT was first developed in 2007 by Pluye et al. [28] and was revised in 2011 by Pace et al. [29] and more recently by Hong et al. [27]. The MMAT can be used to appraise the quality of primary research based on experiment, observation, or simulation [27–29]. Critical appraisal involves judgment making; therefore, two reviewers will independently carry out the appraisal process. For each article included, a criterion based on the methodology will be implored to appraise the article. Calculation of the overall percentage quality score will be done. The scores will be graded ranging from ≤ 50%, which will be regarded as low quality; 51–75%, which will be average quality; and 76–100%, which will be regarded to be of high quality.

### Ethical considerations

This study will not include human or animal participants; therefore, it does not require ethical approval.

## Discussion

The WHO review recommended male-targeted health interventions to help improve men’s health outcomes [16]. However, a 2010 study by Higgins et al. revealed that men receive considerably less attention [30]. Shand et al. report than men are less willing to engage voluntarily in health education and treatment programs [31]; therefore, health education programs have to be strategically crafted to successfully engage men. The proposed scoping review will be conducted as a first part of a study aimed at adaptation of a health education program to increase uptake of HIV testing services among men in LMICs. The scoping review will be conducted as a first part of a study on adaptation of a health education program to improve uptake of HIV self-testing among men in Rwanda.

In this scoping review, we will include evidence published from the year 2000 as most African countries, which constitute a large number of LMICs, had not fully gained independence before then and any health education programs implemented before 2000 might have been biased toward a certain small portion of the population. For the abovementioned context, we will exclude studies reporting evidence from high-income countries. Furthermore, studies reporting evidence on health education programs for women and children will not be considered for this review.

We anticipate to find relevant studies reporting on health education programs for men in LMICs. The findings of this review may be of importance to policymakers involved in crafting health education programs to improve health outcomes. Furthermore, the findings will guide further implementation research on health education programs for men in LMICs. This review will be disseminated electronically or in print and presented at scientific conferences on health education promotion.

## Data Availability

All data generated or analyzed during this study will be included in the published scoping review article.

## Abbreviations

LMICs: Low- to middle-income countries
MMAT: Mixed methods appraisal tool
WHO: World Health Organization

## References

[1] Baker P, Dworkin SL, Tong S, Banks I, Shand T, Yamey G (2014). The men’s health gap: men must be included in the global health equity agenda. Bulletin of the World Health Organization..

[2] Jamison Dean T, Summers Lawrence H, Alleyne George, Arrow Kenneth J, Berkley Seth, Binagwaho Agnes, Bustreo Flavia, Evans David, Feachem Richard G A, Frenk Julio, Ghosh Gargee, Goldie Sue J, Guo Yan, Gupta Sanjeev, Horton Richard, Kruk Margaret E, Mahmoud Adel, Mohohlo Linah K, Ncube Mthuli, Pablos-Mendez Ariel, Reddy K Srinath, Saxenian Helen, Soucat Agnes, Ulltveit-Moe Karen H, Yamey Gavin (2013). Global health 2035: a world converging within a generation. The Lancet.

[3] Wang Haidong, Dwyer-Lindgren Laura, Lofgren Katherine T, Rajaratnam Julie Knoll, Marcus Jacob R, Levin-Rector Alison, Levitz Carly E, Lopez Alan D, Murray Christopher JL (2012). Age-specific and sex-specific mortality in 187 countries, 1970–2010: a systematic analysis for the Global Burden of Disease Study 2010. The Lancet.

[4] Skovdal M, Campbell C, Madanhire C, Mupambireyi Z, Nyamukapa C (2011). Gregson SJG, et al. Masculinity as a barrier to men’s use of HIV services in Zimbabwe..

[5] Möller-Leimkühler AMJJoad. Barriers to help-seeking by men: a review of sociocultural and clinical literature with particular reference to depression. 2002;71(1-3):1-9. 10.1016/s0165-0327(01)00379-2 12167495

[6] MOHSS. National strategic framework for HIV and AIDS response in Namibia 2017/18 to 2021/22. Namibia; 2017. https://www.unaids.org/sites/default/files/country/documents/NAM_2018_countryreport.pdf.

[7] Health Mo. Fourth health sector strategic plan July 2018 - June 2024. Kigali, Rwanda; 2018. http://moh.gov.rw/fileadmin/templates/Docs/FINALH_2-1.pdf.

[8] Mills EJ, Beyrer C, Birungi J (2012). Dybul MRJPm. Engaging men in prevention and care for HIV/AIDS in Africa..

[9] Conserve Donaldson F., Muessig Kathryn E., Maboko Leonard L., Shirima Sylvia, Kilonzo Mrema N., Maman Suzanne, Kajula Lusajo (2018). Mate Yako Afya Yako: Formative research to develop the Tanzania HIV self-testing education and promotion (Tanzania STEP) project for men. PLOS ONE.

[10] Bogle V (2013). A review of the literature: men’s health-seeking behaviour and use of the internet.

[11] Hubley J (1980). Community development and health education. Journal of the Institute of Health Education..

[12] Lee Bandy X., Kjaerulf Finn, Turner Shannon, Cohen Larry, Donnelly Peter D., Muggah Robert, Davis Rachel, Realini Anna, Kieselbach Berit, MacGregor Lori Snyder, Waller Irvin, Gordon Rebecca, Moloney-Kitts Michele, Lee Grace, Gilligan James (2016). Transforming Our World: Implementing the 2030 Agenda Through Sustainable Development Goal Indicators. Journal of Public Health Policy.

[13] Wang H, Naghavi M, Allen C, Barber RM, Bhutta ZA, Carter A, et al. Global, regional, and national life expectancy, all-cause mortality, and cause-specific mortality for 249 causes of death, 1980–2015: a systematic analysis for the Global Burden of Disease Study 2015. 2016;388(10053):1459-544. 10.1016/S0140-6736(16)31012-1 PMC5388903 27733281

[14] Elterman DS, Pelman RS (2014). Male health: a new paradigm, strategies for care delivery, advocacy, education and research. Revista Médica Clínica Las Condes..

[15] Ghanotakis E, Hoke T, Wilcher R, Field S, Mercer S, Bobrow EA (2017). Evaluation of a male engagement intervention to transform gender norms and improve family planning and HIV service uptake in Kabale. Uganda. Global Public Health..

[16] Barker G, Ricardo C, Nascimiento M. Engaging men and boys in changing gender-based inequity in health: evidence from programme interventions. Geneva: World Health Organization; 2007. http://www.who.int/gender/documents/Engaging_men_boys.pdf.

[17] Davis J, Luchters S, Holmes W. Men and maternal and newborn health: benefits, harms, challenges and potential strategies for engaging men. Melbourne: Compass: Women’s and Children’s Health Knowledge Hub. 2012. http://www.mencare.org/data/Men%20and%20Maternal%20and%20Newborn%20Health%20-%20Australia.pdf.

[18] Montgomery EB, Lieberman A, Singh G, Fries JF (1994). Patient education and health promotion can be effective in Parkinson’s disease: a randomized controlled trial. The American Journal of Medicine..

[19] Kirby D (2009). Laris BJCDP. Effective curriculum-based sex and STD/HIV education programs for adolescents..

[20] Morales A, Garcia-Montaño E, Barrios-Ortega C, Niebles-Charris J, Garcia-Roncallo P (2019). Abello-Luque D, et al. Adaptation of an effective school-based sexual health promotion program for youth in Colombia..

[21] Clarke Malcolm, Fursse Joanna, Brown-Connolly Nancy E., Sharma Urvashi, Jones Russell (2018). Evaluation of the National Health Service (NHS) Direct Pilot Telehealth Program: Cost-Effectiveness Analysis. Telemedicine and e-Health.

[22] Colquhoun HL, Levac D, O'Brien KK, Straus S, Tricco AC, Perrier L (2014). Scoping reviews: time for clarity in definition, methods, and reporting. Journal of Clinical Epidemiology..

[23] Arksey H, O’Malley L (2005). Scoping studies: towards a methodological framework. International Journal of Social Research Methodology..

[24] Levac D, Colquhoun H, O’Brien KK (2010). Scoping studies: advancing the methodology. Implementation Science..

[25] Fantom N, Serajuddin U (2016). The World Bank’s classification of countries by income: The World Bank.

[26] Tricco AC, Lillie E, Zarin W, O'Brien KK, Colquhoun H, Levac D (2018). PRISMA extension for scoping reviews (PRISMA-ScR): checklist and explanation. Annals of internal medicine..

[27] Hong Q, Pluye P, Fàbregues S, Bartlett G, Boardman F, Cargo M, Dagenais P, Gagnon MP, Griffiths F, Nicolau B, et al. Mixed methods appraisal tool (MMAT), version 2018. Montréal: McGill; 2018. 10.1016/j.jclinepi.2019.03.008 30905698

[28] Pluye P, Gagnon MP, Griffiths F, Johnson-Lafleur J (2009). A scoring system for appraising mixed methods research, and concomitantly appraising qualitative, quantitative and mixed methods primary studies in Mixed Studies Reviews. International journal of nursing studies..

[29] Pace R, Pluye P, Bartlett G, Macaulay AC, Salsberg J, Jagosh J (2012). Testing the reliability and efficiency of the pilot Mixed Methods Appraisal Tool (MMAT) for systematic mixed studies review. International journal of nursing studies..

[30] Higgins Jenny A., Hoffman Susie, Dworkin Shari L. (2010). Rethinking Gender, Heterosexual Men, and Women's Vulnerability to HIV/AIDS. American Journal of Public Health.

[31] Shand Tim, Thomson-de Boor Hayley, van den Berg Wessel, Peacock Dean, Pascoe Laura (2014). The HIV Blind Spot: Men and HIV Testing, Treatment and Care in Sub-Saharan Africa. IDS Bulletin.

